# The influence of caffeine ingestion on strength and power performance in female team-sport players

**DOI:** 10.1186/s12970-016-0157-4

**Published:** 2016-12-05

**Authors:** Ajmol Ali, Jemma O’Donnell, Andrew Foskett, Kay Rutherfurd-Markwick

**Affiliations:** 1 School of Sport and Exercise, Massey University, Auckland, New Zealand; 2 School of Food and Nutrition, Massey University, Auckland, New Zealand

**Keywords:** Supplementation, Pharmacological ergogenic aid, Muscle function, Knee flexors, Knee extensors

## Abstract

**Background:**

The aim of this study was to examine the influence of caffeine supplementation on knee flexor and knee extensor strength before, during and after intermittent running exercise in female team-sport players taking oral contraceptive steroids (OCS).

**Method:**

Ten healthy females (24 ± 4 years; 59.7 ± 3.5 kg; undertaking 2–6 training sessions per week) taking low-dose monophasic oral contraceptives of the same hormonal composition took part in a randomised, double-blind, placebo-controlled crossover-design trial. Sixty minutes following the ingestion of a capsule containing 6 mg∙kg^−1^ body mass anhydrous caffeine or artificial sweetener (placebo), participants completed a 90-min intermittent treadmill-running protocol. Isometric strength performance and eccentric and concentric strength and power of the knee flexors and knee extensors (using isokinetic dynamometer), as well as countermovement jump (CMJ), was measured before, during and after the exercise protocol, as well as ~12 h post-exercise. Blood samples were taken before, during and post-exercise to measure glucose, insulin and free fatty acids (FFA).

**Results:**

Caffeine supplementation significantly increased eccentric strength of the knee flexors (*P* < 0.05) and eccentric power of both the knee flexors (*P* < 0.05) and extensors (*P* < 0.05). However, there was no effect on isometric or concentric parameters, or CMJ performance. FFA was elevated with caffeine supplementation over time (*P* < 0.05) while levels of glucose and insulin were not affected by caffeine intake.

**Conclusion:**

Caffeine supplementation increased eccentric strength and power in female team-sport players taking OCS both during an intermittent running protocol and the following morning.

## Background

Caffeine is used as an ergogenic aid in both endurance exercise [1] and team-sports [2]. In resistance exercise, the effects are less clear, although supplementation appears to increase peak strength of the knee extensors [3] and time to fatigue in submaximal isometric contractions [4, 5]. Supplementation has also been shown to decrease feelings of fatigue and promote mood and perceptual responses during exercise [6, 7].

Lower body muscle strength and power are critical components of performance in team sports like soccer [8]. Although the use of caffeine is similar in both male and female sports players [9], caffeine supplementation studies have primarily focused on males or a mixed-gender population and little is known about the effects of caffeine on muscular performance specifically in women [3, 10]. In males, despite meta-analytical outcomes showing a small but positive effect of caffeine on knee extensor strength, there is variation among studies in terms of effect size, participants and protocols used [3, 10] so that individual studies show equivocal results. When caffeine supplementation has shown a significant effect on leg muscle strength, this appears primarily in the knee extensors, as opposed to the knee flexors and smaller muscle groups [3]. While initial studies reported the enhancement of peak torque for isometric exercise only [11, 12], recent work has shown a caffeine-induced increase in knee extensor strength regardless of contraction mode [13]. The effect of caffeine intake on power output is unclear [2, 10]. For example, 5 mg · kg^−1^ caffeine produced a lower sprint time in physically active men [14] and 6 mg · kg^−1^ improved sprint speed and drive power in male rugby players [15]. However, no effect on performance was seen in male team-sport athletes during 20 m sprints following ingestion of 6 mg · kg^−1^ caffeine [16]. Research on muscular performance has predominantly focused on isometric or concentric movement [3, 17] with less information on eccentric muscle action. In addition, research determining the effects of caffeine supplementation on leg strength variables in team-sport athletes has often been conducted in the absence of ‘background’ running [18, 19] seen in the prolonged intermittent exercise characteristic of team-sports, limiting its application to performance in competition.

Caffeine supplementation in women is complicated by the effects of both estrogen and oral contraceptive steroids (OCS) on caffeine metabolism [20]; both appear to extend the half-life of caffeine thereby prolonging its effects in the body. Menstrual cycle phase has also been shown to impact strength training outcomes [21]. Of the few caffeine supplementation studies in women most have been carried out without consideration for variation in performance due to menstrual cycle phase [22–24].

We have recently shown that the elimination half-life of caffeine is significantly extended in female team-sport players using OCS [25]. Caffeine supplementation also decreased fatigue and promoted feelings of pleasure during intermittent exercise in this cohort [7]. While it is likely that caffeine supplementation and OCS-associated metabolism have important implications for performance in women undertaking high-impact sport, this information is currently lacking. Thus, research determining the effect of caffeine supplementation on strength and power variables during intermittent exercise in female team-sport players using OCS is necessary. We previously showed ingestion of caffeine prior to an evening intermittent exercise protocol decreased sleep quality parameters, despite an absence of adverse effects upon waking [25]. Since lack of sleep has been associated with decreased performance [26], it is essential to determine the effect of caffeine supplementation on strength and power in women taking OCS the day following ingestion and subsequent sleep impairment.

Therefore, the aim of the current study was to explore the influence of evening caffeine ingestion on knee extensor and knee flexor strength and power during and following intermittent exercise in female team-sport players taking OCS. We hypothesised that, compared to placebo, caffeine supplementation would increase the peak strength and power of the knee flexors and extensors throughout intermittent exercise designed to simulate a soccer game.

## Methods

This research formed part of a larger study reporting the effects of caffeine ingestion on sleep quality [25] and perceptual and cognitive responses [7] in female games players. Therefore only methods specific to the current dataset will be described in detail here. Plasma caffeine levels in response to acute caffeine ingestion are presented in Ali et al. [25].

### Participants

Ten female (mean ± SD; 24 ± 4 years; 59.7 ± 3.5 kg; maximal oxygen uptake 50.0 ± 5.3 ml∙kg^−1^∙min^−1^) team sports players (soccer, hockey and netball), who trained 2-6 times per week and competed at recreational to international level, volunteered for the study. Prior to taking part, participants were fully informed of the study requirements, benefits and risks, and written consent was provided. All procedures had prior approval by the Massey University Human Ethics Committee (Southern A 10/01). Height was measured using a stadiometer and body mass using scales accurate to 0.1 kg (A & D Weighing, HV-200KGL, Australia).

### Experimental controls

Caffeine-naïve participants (who actively refrained from consuming caffeine-containing products) and those with a high daily intake (more than four cups of coffee per day) of caffeine were excluded from the study. Among the participants, self-reported daily caffeine intake varied from 0 to 300 mg∙day^−1^. While not all participants consumed caffeine products every day, all indicated consumption of caffeine-containing products on a weekly basis. All participants were required to keep a food diary for 48-h before their first main trial and replicate this diet before their second main trial. Foodworks (version 6.0.2562, 2009, Xyris Software) was used for the analysis of food diaries. There was no difference in carbohydrate (*P* = 0.55), fat (*P* = 0.91), protein (*P* = 0.19) or overall energy intake (*P* = 0.11) between trials. During the 48-h period, alcohol and foods containing caffeine (chocolate, tea, coffee, soft-drinks, and energy drinks) were to be avoided. Participants were also required to refrain from exercise for the final 24 h of the 48-h period as well as fast for 3 h before the main trial, with only water consumption allowed.

All participants had been taking a monophasic oral contraceptive (Monofeme, Microgynon, Levlen ED or Nordette) of the same hormonal composition (30 μg Ethinyloestradiol and 150 μg Levonorgestrel) for at least 3 months prior to the study. All testing was performed during days 5–8 and 18–22 of one pill-cycle to rule out hormonal influences on changes in energy metabolism or high-intensity intermittent exercise performance [27]. Participants were asked to keep a daily record of their oral contraceptive use, with timing between trials as consistent as possible.

### Blood sampling and analysis

Blood samples were taken at rest (pre), every 15 min during exercise, immediately post-exercise and 12 ± 2 h post-exercise by insertion of an 18-gauge, 1.3-mm intravenous cannula (reference 381244, Insyte, Becton Dickson, NJ, USA) into a medial antecubital vein. Regular flushing with saline (0.9% sodium chloride, Demo S.A. Pharmaceutical Industry, Athens, Greece) kept the cannula patent. Four millilitre blood samples were collected in EDTA tubes, centrifuged for 10 min at 3500 rpm and stored at -80 °C.

FFA concentrations were determined based on the ACS-ACOD method (Wako Pure Chemical Industries, Ltd. Osaka, Japan). Insulin concentrations were determined using radioimmunometric assay (IBL International GMBH, Tecan, Hamburg, Germany, IBMG13021).

### Experimental trials

**Fig. 1:**
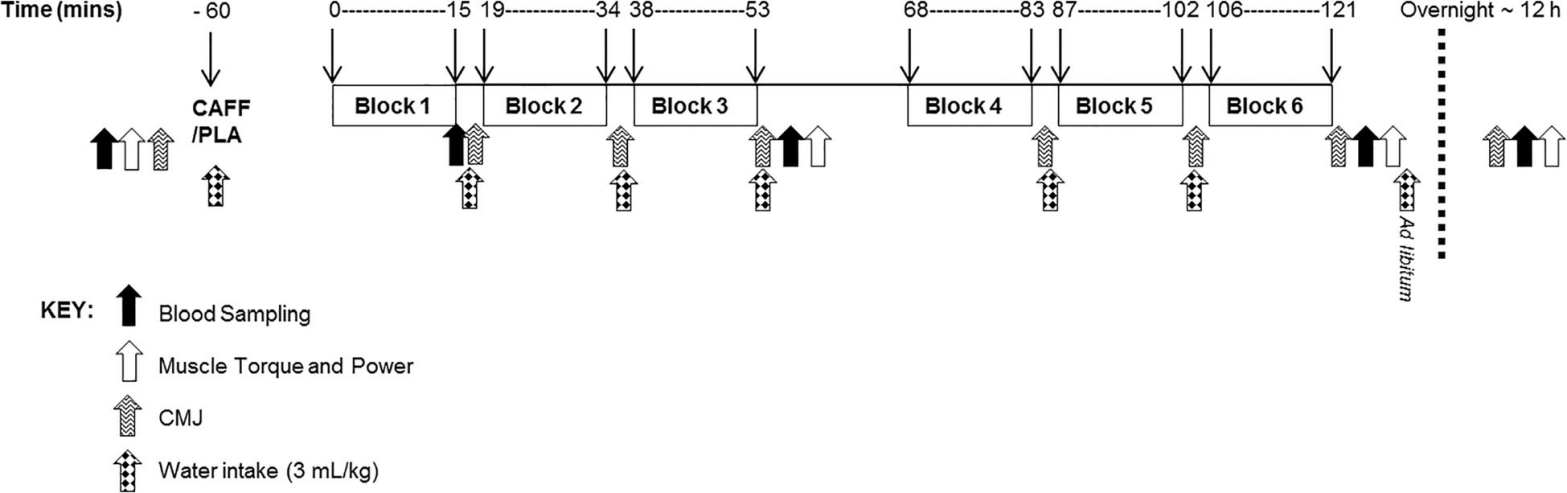
Schematic representation of protocol. Intermittent exercise was carried out in six 15-min blocks 60 min after ingestion of caffeine (CAFF) or placebo (PLA). Plasma concentrations of insulin and free fatty acids were assessed following blood sampling (black arrows), leg muscle strength and power were determined using an isokinetic dynamometer (eccentric and concentric knee extensors and flexors, white arrows) and counter movement jump (CMJ) height was used as a concurrent measure of power (hashed arrows). Final measurements were taken the following day, approximately 12 h after the exercise protocol

Participants were required to perform one familiarisation session involving an incremental VO_2_ max test on a treadmill, followed by two 15-min blocks of the intermittent treadmill running protocol [28] and each dynamometer-associated contraction before commencing the main trials. Expiratory gas collection was performed using the PowerLab metabolic system (8 M, Model ML870, AD Instruments, Australia) and analysis of the expired air for CO_2_ and O_2_ concentrations was performed using a gas analyser (Servomex, Model ML206, AD Instruments, Australia). Participants returned to the lab during days 5–8 and 18–22 of their OCS cycle to begin main trials. Treatments were randomly assigned using a placebo-controlled, double-blind crossover design. On arrival, participants had a cannula inserted and a blood sample taken, then ingested a whole gelatin capsule (Vegie Capsules, BioBalance, New Zealand) containing either 6 mg∙kg^−1^ anhydrous caffeine (Fluka Sigma-Aldrich, St Louis, MO) or placebo (artificial sweetener, Equal) with a 500-mL bolus of water. The intermittent exercise protocol (Fig. 1) began 60 min following ingestion and consisted of six 15-min blocks of treadmill running designed to simulate the activity patterns of a soccer match [28]. Each block consisted of six 2-min running stages at speeds corresponding to 40, 60, 80, 40, 60, and 80% of VO_2_ max, followed by 1 min at 60% VO_2_ max and a 2-min walk (4 km∙h^−1^). Heart rate was monitored and recorded throughout the VO_2_ max test and main trials, using short range telemetry (Polar RS400, Kempele, Finland). Data was downloaded using the Polar ProTrainer computer software (version 5.35.160, Finland). Hydration status was assessed by urine specific gravity before caffeine ingestion, and 12 h post-exercise, using a hand-held refractometer (Sur-Ne, Atago Co. Ltd., Japan).

### Strength and power testing

Leg strength and power were assessed using an isokinetic dynamometer (model 850–230, Biodex Medical Systems Inc., New York). Participants performed five reciprocal concentric contractions of the knee extensors and flexors at 30° · s^−1^ which was repeated using eccentric contractions. Isometric contractions using the knee flexors and extensors with the joint angle at the knee set at 75° were then carried out. The four protocols were programmed to be performed one after the other, with little or no rest period due to time constraints. All participants used their dominant leg and received verbal encouragement and visual feedback as displayed by the Biodex computer. Peak torque was defined as the highest value achieved across the five contractions. Average power for isokinetic concentric and eccentric contractions of the knee flexors and extensors was calculated by the Biodex software (Biodex Advantage Software, V4X). Calibration was performed before each test according to the Biodex setup protocol, in which angles for joint range of motion were individually set.

### Countermovement jump

The countermovement jump (CMJ) was performed using a jump mat (Just Jump System 7610, Perform Better, USA). Participants kept their hands on their hips to minimise momentum generated from arm and upper body movements. An estimate of leg power was calculated from jump height [29]:

$$ Power\ (W) = 60.7\ x\  jump\  height\ (cm) + 45.3\ x\  body\  mass\ (kg) - 2055 $$

### Statistical analysis

Data were compared using two-way analysis of variance (ANOVA) with repeated measures (SPSS version 19.0. Chicago, IL) to examine main effects of i) treatment and ii) time as well as iii) interaction of treatment x time. Mauchly’s test for sphericity was applied to the data. When sphericity was violated the Huynh-Feldt estimate was used to correct the data. When significant differences between the interventions were identified by ANOVA, post-hoc Student’s *t*-test, using the Holm-Bonferroni adjustment, were performed. Data is presented as mean ± SD. Statistical significance was set at *P* < 0.05 whereas ‘practical’ significance was assessed using Cohen’s d values. Effect size cut-offs were defined as 0.2, 0.5 and 0.8 for small, medium and large effect sizes, respectively.

## Results

### Muscle strength

**Fig. 2:**
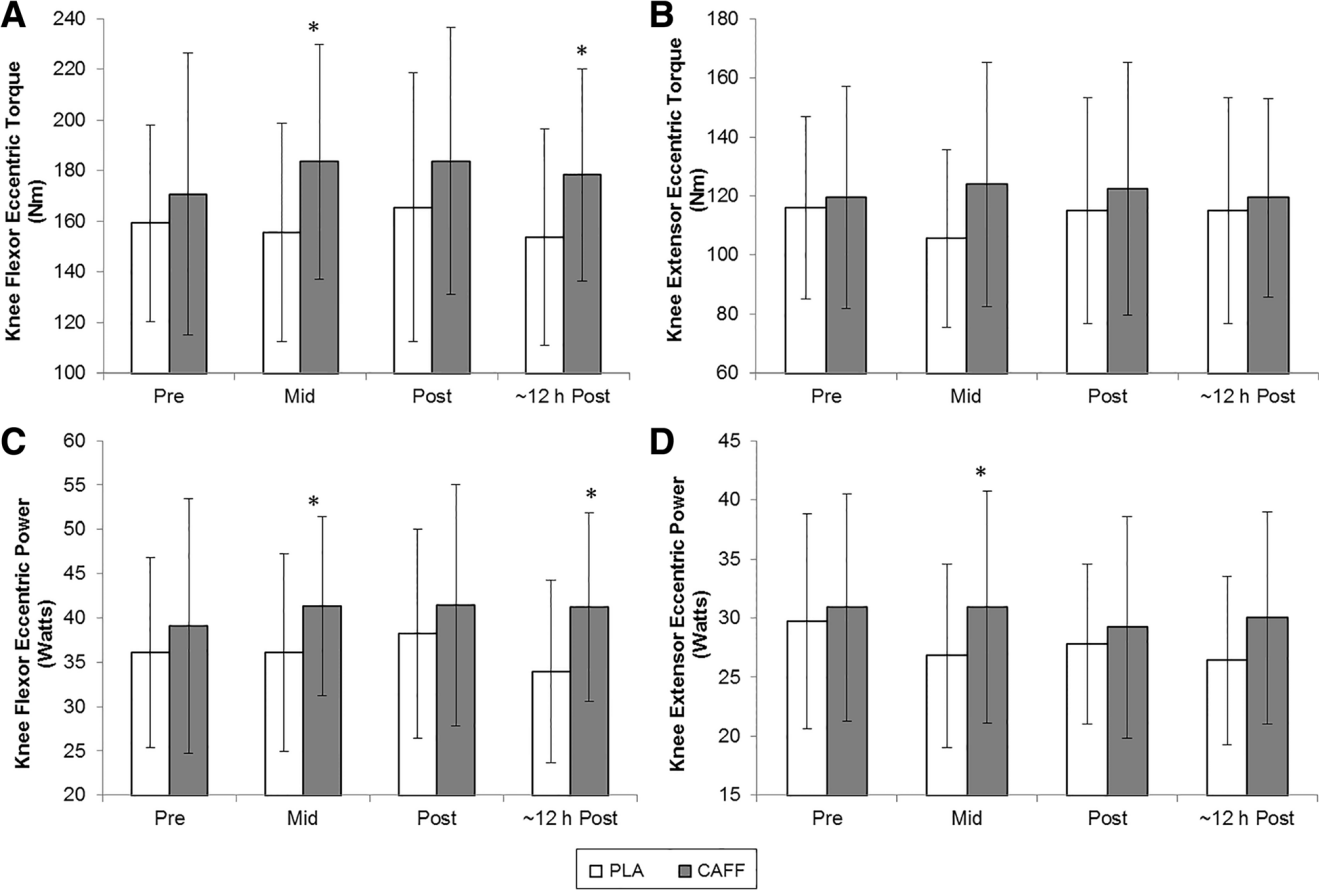
Mean (± SD) eccentric knee flexor (**a** and **c**) and extensor (**b** and **d**) strength (**a** and **b**) and power (**c** and **d**) parameters for caffeine (CAFF) and placebo (PLA) trials before, during and after the 90-min intermittent exercise protocol. * Significantly higher in CAFF trial (*P* < 0.05; *n* = 10)

Caffeine ingestion increased peak torque values for the knee flexors during eccentric contractions (*P* = 0.024; Cohen’s d = 0.45, Fig. 2a). There was no effect of caffeine ingestion on peak torque for the knee extensors during eccentric contraction at any stage in the trial (*P* = 0.291, Cohen’s d = 0.24, Fig. 2b).

Peak torque values were not significantly different between caffeine and placebo trials for knee flexors during concentric contractions (*P* = 0.327, Cohen’s d = 0.11, Table 1). There was a trend for caffeine ingestion to increase knee extensor strength (*P* = 0.053; Cohen’s d = 0.29). There was no effect of caffeine on isometric strength (Table 2) in the knee extensors (*P* = 0.837; Cohen’s d = 0.04) or knee flexors (*P* = 0.143; Cohen’s d = 0.18).

**Table 1:** Mean (± SD) concentric knee flexor and extensor strength and power parameters for caffeine (CAFF) and placebo (PLA) trials before, during and after the 90-min intermittent exercise protocol (*n* = 10)

	Sample time					Cohen’s d (Trial Mean)	Statistical significance (*P* values)		
	Pre	Mid	Post	~12 h post	Trial Mean		Treatment	Time	Treatment x Time
Average torque (Nm)									
*Concentric knee flexor*									
PLA	79.7 ± 21.2	77.9 ± 23.4	77.1 ± 20.8	76.0 ± 18.8	77.6 ± 20.3	0.11	0.327	0.920	0.222
CAFF	75.9 ± 19.8	80.6 ± 17.3	80.2 ± 20.1	82.4 ± 19.1	79.8 ± 18.5				
*Concentric knee extensor*									
PLA	104.4 ± 34.6	105.0 ± 35.7	111.8 ± 43.2	102.6 ± 32.7	106.0 ± 35.5	0.29	0.053	0.678	0.257
CAFF	112.6 ± 45.3	115.7 ± 33.7	114.4 ± 32.3	124.2 ± 43.2	116.8 ± 37.8				
Average power (Watts)									
*Concentric knee flexor*									
PLA	25.7 ± 7.5	23.7 ± 6.5	24.0 ± 7.1	24.6 ± 6.2	24.5 ± 6.6	0.11	0.274	0.076	0.385
CAFF	24.8 ± 7.1	24.5 ± 7.4	24.5 ± 5.8	26.9 ± 6.8	25.2 ± 6.6				
*Concentric knee extensor*									
PLA	24.3 ± 8.7	23.8 ± 9.0	25.5 ± 9.2	23.5 ± 7.6	24.3 ± 8.4	0.23	0.151	0.372	0.069
CAFF	23.1 ± 8.0	26.7 ± 8.0	27.0 ± 8.1	28.2 ± 9.6	26.2 ± 8.3				

Cohen’s d values are based on the corresponding trial mean

**Table 2:** Mean (± SD) isometric knee flexor and extensor strength parameters for caffeine (CAFF) and placebo (PLA) trials before, during and after the 90-min intermittent exercise protocol (*n* = 10)

Average torque (Nm)	Sample time					Cohen’s d (Trial Mean)	Statistical significance (*P* values)		
	Pre	Mid	Post	~ 12 h post	Trial Mean		Treatment	Time	Treatment x Time
*Isometric knee flexor*									
PLA	77.6 ± 18.6	77.0 ± 16.9	76.3 ± 17.5	76.1 ± 19.1	76.7 ± 17.3	0.18	0.143	0.981	0.841
CAFF	77.9 ± 18.3	79.9 ± 24.9	81.7 ± 17.0	80.8 ± 22.4	80.1 ± 20.1				
*Isometric knee extensor*									
PLA	157.4 ± 40.8	155.3 ± 38.9	156.1 ± 35.1	154.2 ± 42.3	155.7 ± 37.8	0.04	0.837	0.845	0.657
CAFF	153.6 ± 47.7	151.8 ± 30.0	159.9 ± 44.0	164.6 ± 48.9	157.4 ± 42.0				

Cohen’s d values are based on the corresponding trial mean

### Muscle power

Mean power, as determined over the 5 contractions for each participant per sampling point, was significantly improved with caffeine ingestion during eccentric contractions of the knee flexors (*P* = 0.029; Cohen’s d = 0.42, Fig. 2c) and eccentric contractions of the knee extensors (*P* = 0.040; Cohen’s d = 0.30, Fig. 2d). While the latter decreased over time (*P* = 0.032) there was no interaction of treatment and time (*P* = 0.250). For concentric contraction, there were no significant effects of caffeine intake on mean power in the knee extensors (*P* = 0.151; Cohen’s d = 0.23) or knee flexors (*P* = 0.274, Cohen’s d = 0.11; Table 1).

There was no effect of caffeine intake on CMJ height (*P* = 0.582, Cohen’s d = 0.08, Table 3), or estimated leg power based on CMJ height (*P* = 0.391, Cohen’s d = 0.09, Table 3). However, both CMJ height (*P* < 0.001) and estimated leg power (*P* < 0.001) decreased significantly over time. Post-hoc testing revealed that the differences lay between the pre-exercise value and the subsequent measures after each block of exercise.

**Table 3:** Mean (± SD) counter-movement jump (CMJ) and estimated power parameters for caffeine (CAFF) and placebo (PLA) trials before, during and after the 90-min intermittent exercise protocol (*n* = 10)

	Sample time									Cohen’s d (Trial Mean)	Statistical significance (*P* values)		
	Pre	Block 1	Block 2	Block 3	Block 4	Block 5	Block 6	~12 h post	Trial Mean		Treatment	Time	Treatment x Time
*CMJ Height (cm)*													
PLA	38.4	40.8	40.5	40.7	41.6	39.9	39.4	36.2	39.7	0.08	0.582	<0.001	0.951
	± 4.4	± 5.8	± 5.7	± 6.3	± 7.0	± 5.4	± 6.0	± 4.5	± 5.7				
CAFF	38.5	41.6	40.8	41.0	41.0	39.8	40.5	37.2	40.1				
	± 5.5	± 4.7	± 4.1	± 5.4	± 4.3	± 4.0	± 4.2	± 4.9	± 4.7				
*Estimated CMJ Power (Watts)*													
PLA	2976.1	3123.0	3105.7	3116.4	3168.9	3067.1	3039.4	2843.5	3055.0	0.09	0.391	<0.001	0.950
	± 339.2	± 412.0	± 414.2	± 452.5	± 477.6	± 363.1	± 463.9	± 332.2	± 403.7				
CAFF	2996.2	3182.7	3136.5	3144.2	3148.8	3075.2	3116.7	2914.5	3089.3				
	± 377.8	± 354.1	± 305.9	± 377.2	± 314.8	± 278.5	± 321.0	± 372.8	± 390.8				

Cohen’s d values are based on the corresponding trial mean

### Metabolic parameters

**Fig. 3:**
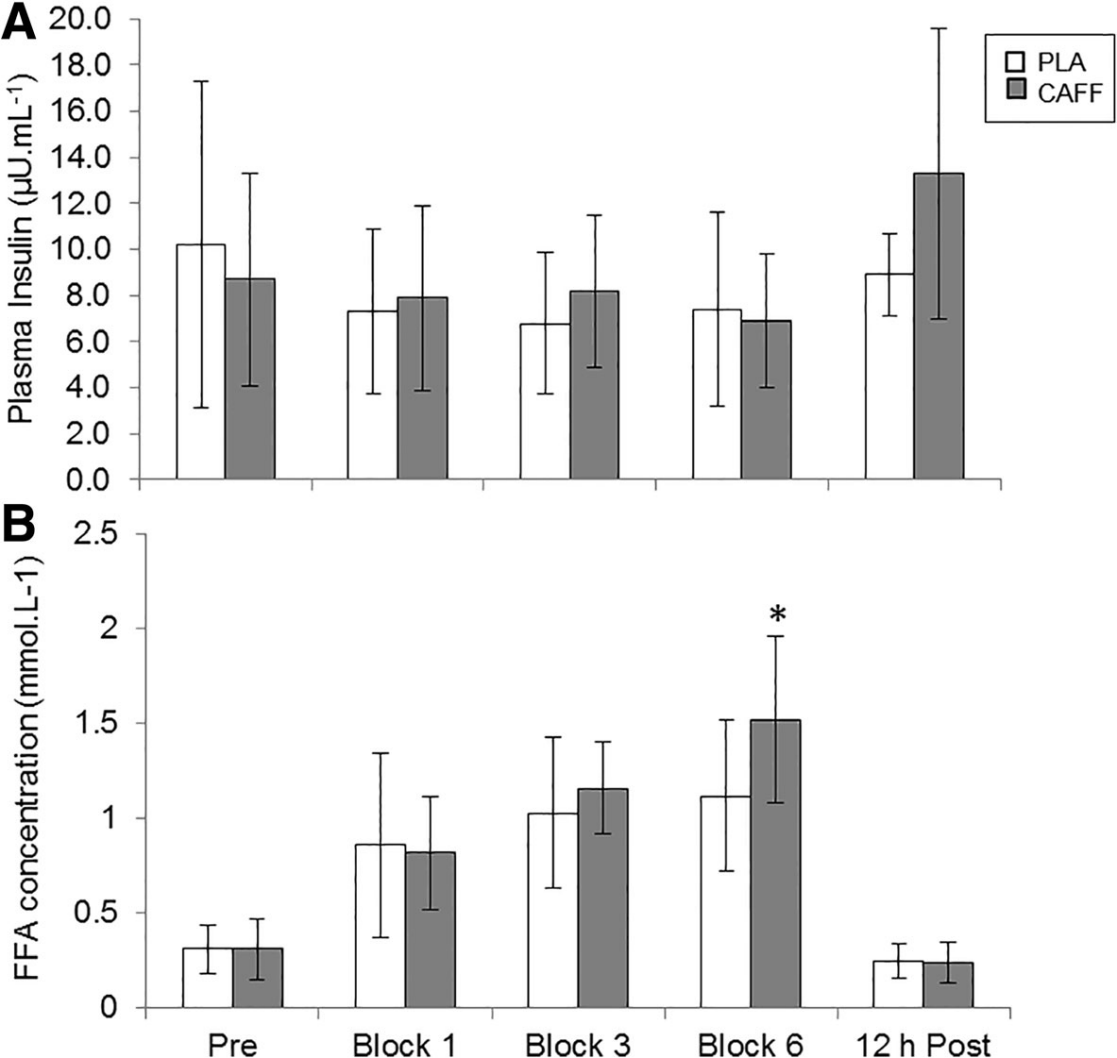
Mean (± SD) plasma concentrations of (**a**) insulin (μU·mL^−1^) and (**b**) free fatty acids (FFA; mmol·mL^−^^1^) for caffeine (CAFF) and placebo (PLA) trials before, during and after the 90-min intermittent exercise protocol. * Significantly higher in CAFF trial (*P* < 0.05; *n* = 10)

Consistent with plasma glucose concentrations [7], plasma insulin concentrations were unaffected by caffeine supplementation (*P* = 0.276; Fig. 3a). Likewise, there was no main effect of treatment for FFA concentration (*P* = 0.311; Fig. 3b). However, there was an interaction effect of treatment x time for FFA concentration (*P* = 0.044) with higher values in the caffeine trial after 90 min of exercise (*P* < 0.05; Fig. 3b).

### Physiological responses to 90 min treadmill test

There was no effect of caffeine ingestion on heart rate (*P* = 0.158), VO_2_ (*P* = 0.446) or relative exercise intensity (% VO_2_ max, *P* = 0.515). However, these parameters all increased over time (HR, *P* = 0.019; VO_2_ *P* = 0.016; % VO_2_ max, *P* = 0.016). Urine specific gravity (USG) was not different between trials (*P* = 0.608) or between pre-exercise and ~12 h post-exercise (*P* = 0.588).

## Discussion

The primary finding of the current study was that caffeine ingestion significantly enhanced eccentric muscle strength and power in female team-sport players during and following intermittent running exercise. This enhancement was predominantly observed in the knee flexors, with knee extensor improvements for average power only. Furthermore, eccentric knee flexor power and strength remained significantly elevated the following morning, approximately 12 h post-caffeine ingestion. Taken together, these results suggest that evening caffeine supplementation has the potential to promote eccentric knee flexor performance during intermittent exercise both immediately after and the day following ingestion in female team-games players taking OCS.

Leg strength and power are essential components of performance in team sports such as soccer and rugby. In addition to kicking, the dynamic and intermittent nature of these games involves a large amount of sprinting-based activity, which places a marked load on the eccentric capabilities of the knee flexors in particular [30]. It can be suggested therefore that enhancing strength and power of these muscles will lead to increased performance. Indeed, there is a relationship between eccentric leg strength and sprinting performance [8, 30] and recent studies have shown a positive correlation between eccentric [31] and/or hamstring-specific [32] training and subsequent sprinting and/or jumping performance in soccer players. In particular, neuromuscular training incorporating both strength and plyometric activities was found to enhance sprint speed and knee flexibility and strength during the eccentric phase of a jump in female basketball, soccer and volleyball players [33]. The current research showed a significant increase in eccentric knee flexor strength and eccentric knee flexor and extensor power, with ingestion of 6 mg · kg^−1^ of caffeine, in female team-sport players during an intermittent running protocol. Although testing was not carried out during competition itself, the background intermittent running protocol was designed to simulate soccer game activity [28]. Given the established importance of eccentric strength and power for sprint and jump performance during team sports [8, 34] it can be suggested that caffeine ingestion will have a significant positive effect on the competitive performance of female games players. However, more research using appropriate field tests needs to be undertaken to confirm this assertion.

Stuart et al. [15] showed that caffeine ingestion enhanced multiple aspects of team-sport performance including sprint time, sprint agility and drive power during a simulated game in male rugby players. However, caffeine metabolism is affected by both estrogen and OCS use [20]; therefore, these results cannot be used to inform female team sports athletes. A caffeine-containing energy drink improved sprint and jump performance in female rugby players before and during a rugby tournament [23] and soccer players during a simulated game [24]. However these studies did not take into account hormonal effects on caffeine metabolism and did not study the effects of caffeine exclusively. Furthermore, none of the above-mentioned research measured isolated parameters of strength. To date therefore, this is the first time that eccentric knee flexor and extensor muscle strength and power have been assessed in female athletes taking OCS.

A number of studies have reported an absence of an ergogenic effect on leg strength with caffeine intake [17, 18]. When an effect has been reported, this has often occurred in the knee extensors without influencing the knee flexors [3]. However, these studies have primarily focused on concentric movement. Similarly, in the current study, there was no significant increase in concentric strength with caffeine supplementation. While we did see a trend for an increase in concentric knee extensor strength the distinction between concentric and eccentric movement is important, particularly in the knee flexors, as eccentric strength of this muscle group consistently correlates with injury risk in games players [30, 34, 35]. The absence of caffeine-mediated strength and/or power improvements published to date may also be explained by the lack of background running in these studies [17, 18] as it is possible that the ergogenic effect of caffeine on strength and power parameters is only apparent when athletes are fatigued. In support of this statement, we have previously shown that perceived ratings of fatigue were inversely associated with peak plasma caffeine concentration [7].

Interestingly, while eccentric knee flexor and knee extensor power increased, there was no increase in CMJ height with caffeine ingestion. However, CMJ performance is considered predominantly concentric work. Eccentric extensor muscle activation pre-jump has been suggested to play a role in CMJ height while knee flexor muscle activation was not involved [34]. Previous research in female soccer players has shown an increase in CMJ height after ingestion of an energy drink containing only 3 mg · kg^−1^ of caffeine, compared to the same energy drink without caffeine [23]. However, this beverage also contained taurine, L-carnitine and sodium bicarbonate, thus a combinatorial effect cannot be ruled out. This study [23] also did not account for any influences of the menstrual cycle or OCS use on caffeine metabolism.

Decreased eccentric knee flexor strength is a major risk factor for non-contact injury in team sports [35]. In particular, soccer players may be prone to anterior cruciate ligament (ACL) injury due to a decreased eccentric knee flexor to concentric knee extensor ratio [8], a phenomenon that appears even more marked in female players [36]. Females show reduced eccentric knee flexor strength compared to males, a finding that is further complicated by biomechanical factors affecting ligament laxity and dynamic stability. Our finding that caffeine enhances eccentric knee flexor strength in females suggests that caffeine supplementation prior to competition and/or training has the potential to decrease non-contact injury rates. Although our findings are speculative, the results form the basis for similar studies to be conducted in females with caffeine supplementation.

There is a possibility that evening caffeine ingestion will have a negative effect on performance measures the following day due to impaired sleep [25] as lack of sleep quality has been associated with decreased performance [26]. While the participants in the current study reported decreased sleep quality with caffeine ingestion [25], there was no decrease in strength or power in any muscle group or activity the following morning. Moreover, eccentric knee flexor power and strength remained significantly elevated in caffeine participants relative to placebo (Fig. 3d). As caffeine and caffeine metabolite levels were also elevated at this time [25], it is possible that performance will decrease when caffeine has been fully metabolised and/or that prolonged evening caffeine ingestion will result in detrimental performance [25, 26]; further research addressing these suggestions is necessary.

The mechanism by which caffeine produces ergogenic effects for muscular strength and power activity is not well understood, although it is most likely to involve a supraspinally-driven increase in voluntary muscle activation [4, 13]. For endurance exercise, caffeine elicits performance benefits primarily via adenosine receptor antagonism [1]. It was initially suggested that caffeine-associated performance increases during aerobic exercise were due to enhanced FFA oxidation and subsequent sparing of muscle glycogen [1, 3]. This theory has been extensively challenged, particularly for isolated, high intensity activity where fuel-associated increases in performance are unlikely due to the anaerobic nature of the exercise [3]. However, team-sport exercise is characterised by short bursts of anaerobic activity in a background of lower intensity running [34]. Here, we used an intermittent running protocol to mimic the nature of a soccer game. There was no change in plasma levels of glucose [7] or insulin (Fig. 3a); while this potentially rules out a glycogen-sparing effect, the exercise intensity in this study was only 60% of VO_2_ max and higher intensities are likely required to examine this in detail.

We saw a greater increase in FFA over time in the caffeine trial, suggesting a caffeine-mediated enhancement of fat mobilisation that is above exercise-induced levels. Can enhanced FFA mobilisation explain the observed increases in eccentric strength and power in the current study? The caffeine-associated FFA concentration increase was restricted to the end of the exercise protocol, while the most significant increases in eccentric strength and power occurred in the middle of the protocol, in line with peak caffeine concentrations [25]. These performance parameters remained elevated the following morning, where the caffeine metabolite paraxanthine was at its highest measured concentration. We conclude therefore that the observed increases in eccentric performance can most likely be attributed to central nervous system stimulation by caffeine and its metabolites. The significant decrease in fatigue with caffeine supplementation in this cohort [7], and an absence of adverse cognitive effects upon waking despite a lower quality of sleep [7, 25] support this conclusion. Additional mechanisms for caffeine’s ergogenic action during anaerobic exercise may include the catecholamine response and an enhancement of excitation-contraction coupling [37].

## Conclusions

We present the first study detailing the effects of caffeine supplementation on strength and power parameters during an intermittent exercise protocol in female team-sport players taking OCS. Our data provides evidence that caffeine ingestion enhances eccentric knee flexor performance in this cohort. This result has implications for both sprinting and kicking-activities dominated by eccentric muscle contraction, suggesting that caffeine supplementation may enhance performance during competition. Moreover, decreased eccentric knee flexor strength and power correlates with increased risk for ACL tears. As these injuries are predominant in female games players and amplified by fatigue, caffeine supplementation both during training and prior to competition may provide a simple means to reduce non-contact injury in female team sports athletes; however, more research is required to examine these issues further.
